# Immunomodulatory effect of betulin and its derivatives on IL-6 expression in colorectal cancer cell lines and molecular docking insights

**DOI:** 10.3389/fmolb.2025.1704804

**Published:** 2025-11-03

**Authors:** Marcel Madej, Adrianna Halama, Elwira Chrobak, Ewa Bębenek, Joanna Magdalena Gola

**Affiliations:** ^1^ Department of Molecular Biology, Faculty of Pharmaceutical Sciences in Sosnowiec, Medical University of Silesia, Katowice, Poland; ^2^ Silesia LabMed, Centre for Research and Implementation, Medical University of Silesia, Katowice, Poland; ^3^ Department of Organic Chemistry, Faculty of Pharmaceutical Sciences in Sosnowiec, Medical University of Silesia, Katowice, Poland

**Keywords:** colorectal cancer, IL-6, betulin, derivatives, mRNA, protein

## Abstract

**Purpose:**

This study investigated the immunomodulatory and therapeutic potential of betulin and its derivatives (EB5 and ECH147) in colorectal cancer (CRC), focusing on their effects on IL-6 expression at the molecular level and their possible application as diagnostic and therapeutic tools.

**Methods:**

Human CRC cell lines (HT-29, RKO, SW1116) and normal colonocytes (CCD-841CoN) were treated with betulin, EB5, ECH147, cisplatin, and 5-fluorouracil (10 μg/mL) for 2, 8, and 24 h. *IL-6* mRNA levels were measured by RT-qPCR in real time, and IL-6 protein was quantified using a proximity ligation immunoassay. Molecular docking was performed using IL-6 structure (PDB ID: 1ALU). Statistical significance was evaluated using Kruskal–Wallis and *post hoc* rank tests.

**Results:**

IL-6 expression was undetectable in HT-29 and RKO cells, both harboring the *BRAF* ^*V600E*^ mutation. ECH147 and EB5 derivatives significantly decreased IL-6 mRNA and protein levels in SW1116 and CCD-841CoN cells at 8 and 24 h. Molecular docking analysis revealed that ECH147 formed a stable hydrogen bond, suggesting direct binding.

**Conclusion:**

Structural modification of betulin enhances its molecular therapeutic activity, with phosphonate derivative ECH147 showing the strongest decrease in IL-6 levels. These findings suggest that IL-6 downregulation can serve as a molecular biomarker for drug efficacy, while ECH147 represents a promising candidate for targeted molecular therapy in CRC. This dual diagnostic–therapeutic approach highlights the potential of betulin derivatives in advancing precision medicine for IL-6–mediated pathways.

## 1 Introduction

Colorectal cancer (CRC) remains a leading cause of cancer-related morbidity and mortality worldwide. Its pathogenesis involves a complex interplay of genetic, epigenetic, environmental, and immunological factors (Wen et al., 2025). Chronic inflammation plays a critical role in both the initiation and progression of CRC, with interleukin-6 (IL-6) recognized as a key pro-tumorigenic mediator (Lu et al., 2025; Zhou et al., 2025; Turano et al., 2021). This cytokine is involved in several processes, including the proliferation of intestinal epithelial cells, apoptosis, and cellular differentiation. It exerts its effects through two signaling pathways: classic signaling and trans-signaling (Li et al., 2024; Turano et al., 2021).

In classic signaling, the fully active form of IL-6 binds to the membrane-bound IL-6 receptor α (IL-6Rα) on the surface of the target cell, forming the IL-6/IL-6Rα complex. Upon binding, the gp130 glycoprotein dimerizes and becomes phosphorylated, leading to the activation of the JAK and MAPK/ERK kinase pathways (Li et al., 2024; Turano et al., 2021). The outcome of classic IL-6 signaling typically includes cell repair, regeneration, and the induction of apoptosis in abnormal cells. In CRC, IL-6 predominantly acts through trans-signaling. In this pathway, circulating IL-6 binds to the soluble form of the IL-6 receptor (sIL-6R). The IL-6/sIL-6R complex subsequently triggers phosphorylation and activates two key signaling cascades: JAK/STAT3 and PI3K/AKT leading to promote chronic inflammation, epithelial–mesenchymal transition, and tumor angiogenesis (Li et al., 2024). Additionally, elevated serum levels of IL-6 have been shown to correlate with more advanced stages of colorectal cancer, highlighting its significant role in disease progression (Zhou et al., 2025). This insight not only deepens our understanding of CRC pathophysiology but also points toward potential biomarkers and treatment targets. Standard treatments for CRC include surgery and chemotherapy, typically with 5-fluorouracil (5-FU), oxaliplatin, or irinotecan. While effective, these therapies often cause systemic toxicity, highlighting the need for safer, targeted alternatives (Fadlallah et al., 2024; Gila et al., 2025).

Natural compounds have gained attention for their multi-targeted activity and lower toxicity profiles. Betulin, a pentacyclic triterpenoid derived from birch bark, exhibits anti-inflammatory, anticancer and neuroprotective properties (Farzan et al., 2024; El-Sherbiny et al., 2021). It can induce apoptosis, suppress proliferation and metastasis, and modulate inflammation. However, its clinical application is limited by poor solubility and bioavailability. To address this, semi-synthetic derivatives have been developed to enhance its pharmacokinetic profile (Farzan et al., 2024; Nemli et al., 2024).

This study investigates whether betulin and its derivatives modulate IL-6 expression in CRC. The effects of betulin, EB5 (28-propynoylbetulin), and ECH147 (29-diethoxyphosphoryl-28-propynoylbetulin) on IL-6 mRNA and protein levels were evaluated in CRC cell lines SW1116, HT-29, and RKO. Normal colon epithelial cells (CCD-841CoN) were included to assess selectivity. Additionally, molecular docking was performed to explore potential interactions between the compounds and IL-6, providing insight into their mechanisms of action.

## 2 Materials and methods

### 2.1 Cell culture of colorectal cancer cell lines and normal colonocytes

The colorectal cancer cell lines RKO, HT-29, and SW1116 (catalog numbers CRL-2577, HTB-38, and CCL-233, respectively; ATCC, Manassas, VA, United States), along with the normal colon epithelial cell line CCD-841CoN (CRL-1790, ATCC), were cultured under standard *in vitro* conditions at 37 °C in a humidified atmosphere containing 5% CO_2_ using a Direct Heat CO_2_ incubator (ThermoFisher Scientific, Waltham, MA, United States). Each cell line was maintained in its recommended growth medium as per the supplier’s protocol. Specifically, RKO and CCD-841CoN cells were propagated in Eagle’s Minimum Essential Medium (EMEM), HT-29 cells were cultured in McCoy’s 5A medium, and SW1116 cells were grown in Leibovitz’s L-15 medium. All media were supplemented with 10% fetal bovine serum (EuroClone, Milan, Italy) and 50 μg/mL gentamicin (BioWhittaker, Lonza, Basel, Switzerland) to support cell growth and prevent microbial contamination. The main genetic and epigenetic profiles of each CRC cell lines are summarized in Table 1 (Ahmed et al., 2013).

**TABLE 1:** Main genetic and epigenetic characteristics of the HT-29, RKO, SW1116 cell lines.

Cell line	MSI status	CIMP status	CIN status	Gene mutations
RKO	MSI	+	-	*BRAF* ^*V600E*^, *PIK3CA* ^*H1047R*^, *KRAS*-wt, *PTEN*-wt, *TP53*-wt
HT-29	MSS	+	+	*BRAF* ^*V600E*^, *PIK3CA* ^*P449T*^, *KRAS*-wt, *PTEN*-wt, *TP53* ^*R273H*^
SW1116	MSS	+/−	+	*BRAF-wt*, *PIK3CA-wt*, *KRAS* ^*G12A*^, *PTEN*-wt, *TP53* ^*A159D*^

MSI—microsatellite instability; MSS—microsatellite stable; CIMP—CpG island methylator phenotype; CIN—chromosomal instability pathway; wt—wild type.

### 2.2 Treatment strategy and time intervals

The betulin and its derivatives EB5 and ECH147 were kindly provided by the Department of Organic Chemistry at the Faculty of Pharmaceutical Sciences in Sosnowiec, Medical University of Silesia (Katowice, Poland). The synthesis and detailed chemical characterization of EB5 were previously reported by Boryczka et al., while ECH147 was described by Chrobak et al. (Boryczka et al., 2013; Chrobak et al., 2016). To investigate the influence of betulin and its derivatives on IL-6 expression, CRC lines were treated with betulin, EB5, ECH147, as well as reference chemotherapeutics— 5-FU and cisplatin—each at a final concentration of 10 μg/mL. The concentration was selected based on earlier cytotoxicity studies (Madej et al., 2024). Cisplatin and 5-FU served as standard controls due to their clinical relevance in colorectal cancer therapy.

To assess time-dependent effects, cells were exposed to the test substances for 2, 8, and 24 h. Untreated cells served as the negative control. All experiments were performed in triplicate to ensure reproducibility. After incubation, culture media were collected and centrifuged to remove debris, with the supernatants stored for subsequent protein-level analyses. Following medium removal, 500 μL of TRIzol reagent (Sigma-Aldrich, St. Louis, MO, United States) was added to each well to preserve RNA for downstream molecular analyses.

### 2.3 RNA isolation and assessment of purity and concentration

Total RNA was isolated from the treated cell cultures using TRIzol reagent (Sigma-Aldrich, St. Louis, MO, United States), following the manufacturer’s protocol. RNA concentration and purity were assessed using spectrophotometric measurement with the MaestroNano MN-913 device (MaestroGen Inc., Las Vegas, NV, United States) and confirmed by agarose gel electrophoresis. The resulting RNA samples were subsequently utilized for the analysis of gene expression at the mRNA level.

### 2.4 Evaluation of transcriptional alterations using RT-qPCR in real time

The expression levels of the *IL-6* gene were quantified using real-time RT-qPCR performed on the LightCycler® 480 System (Roche, Basel, Switzerland). The GoTaq® 1-Step RT-qPCR kit (Promega, Madison, WI, United States) was employed according to the reaction parameters previously established by Kruszniewska-Rajs et al. (Kruszniewska-Rajs et al., 2022). For amplification of the *IL-6* mRNA transcript, the following primers were used: forward 5′-GCAGAAAAAGGCAAAGAATC-3′ and reverse 5′-CTACATTTGCCGAAGAGC-3′. β-actin (ACTB) functioned as the endogenous control gene. Relative quantification of mRNA expression was calculated using the 2^−ΔΔCT^ method. All reactions were carried out in triplicate to ensure reproducibility. Specificity of the amplification products was confirmed by melting curve analysis as well as by electrophoretic separation on a 2% agarose gel.

### 2.5 Proximity ligation assay-based evaluation of IL-6 protein in cell culture media

IL-6 protein concentrations in the cell culture supernatants were measured using the commercially available Human IL-6 ProQuantum Immunoassay Kit (ThermoFisher Scientific, Waltham, MA, United States), following the manufacturer’s protocol. The assay sensitivity ranges from 0.064 to 10,000 pg/mL, with analytical sensitivity <0.05 pg/mL. Prior to analysis, the samples were prepared as described in a previous study, followed by a twofold dilution (Madej et al., 2025). Quantitative PCR was conducted using the QuantStudio 7 Pro Dx Real-Time PCR System (ThermoFisher Scientific Inc., Waltham, MA, United States), and the resulting data were analyzed through the ProQuantum online platform (apps.thermofisher.com/apps/proquantum, accessed June 30, 2025).

### 2.6 Molecular interaction modeling via docking studies

Molecular docking analyses were carried out for 5-FU, betulin, and its derivatives EB5 and ECH147 to investigate their interactions with the IL-6 protein, used as the molecular target. This *in silico* approach aimed to clarify potential mechanisms of action and assess the binding affinity and specificity between the ligands and the target protein (Martis and Téletchéa, 2025). The chemical structures of all compounds were retrieved from the PubChem database (https://pubchem.ncbi.nlm.nih.gov/, accessed July 23, 2025) in SDF format and converted to mol2 format using ChimeraX version 1.9.

The crystal structure of the IL-6 protein (PDB ID: 1ALU) was obtained from the RCSB Protein Data Bank (https://www.rcsb.org/, accessed July 23, 2025) in PDB format. Prior to docking, the protein model was prepared by removing solvents and irrelevant molecules using ChimeraX 1.9. Additional refinement steps were performed in AutoDock Tools 1.5.7, including the removal of water molecules and addition of hydrogen atoms. The final protein and ligand structures were saved in PDBQT format for docking.

Docking simulations were executed using AutoDock Vina version 1.1.2. To interpret and visualize the docking results, a combination of Discovery Studio 2025, ChimeraX 1.9, and LigPlot + version 2.2 was employed.

### 2.7 Statistical analysis

Statistical analyses were performed using STATISTICA software (version 13.3; Tibco Inc., Palo Alto, CA, United States). All experiments were conducted in triplicate to ensure reproducibility. Qualitative results were visualized with box-and-whisker plots created using JASP (version 0.19.1.0; University of Amsterdam, Amsterdam, Netherlands), while heatmaps were generated with GraphPad Prism 9.0.2 (GraphPad Software, San Diego, CA, United States).

For quantitative variables not following a normal distribution, data were reported as medians with interquartile ranges. The normality of data distribution was assessed using the Shapiro–Wilk test. Group comparisons were conducted using the Kruskal–Wallis test, a non-parametric equivalent of one-way ANOVA. To account for multiple testing across different experimental groups, analyses were performed separately for each time point. Where significant differences were detected, *post hoc* analysis was performed using mean rank comparisons. A *p-*value of less than 0.05 was considered statistically significant.

## 3 Results

### 3.1 Dynamic expression pattern of *IL-6* gene in tested cell lines during compound treatment

**FIGURE 1:**
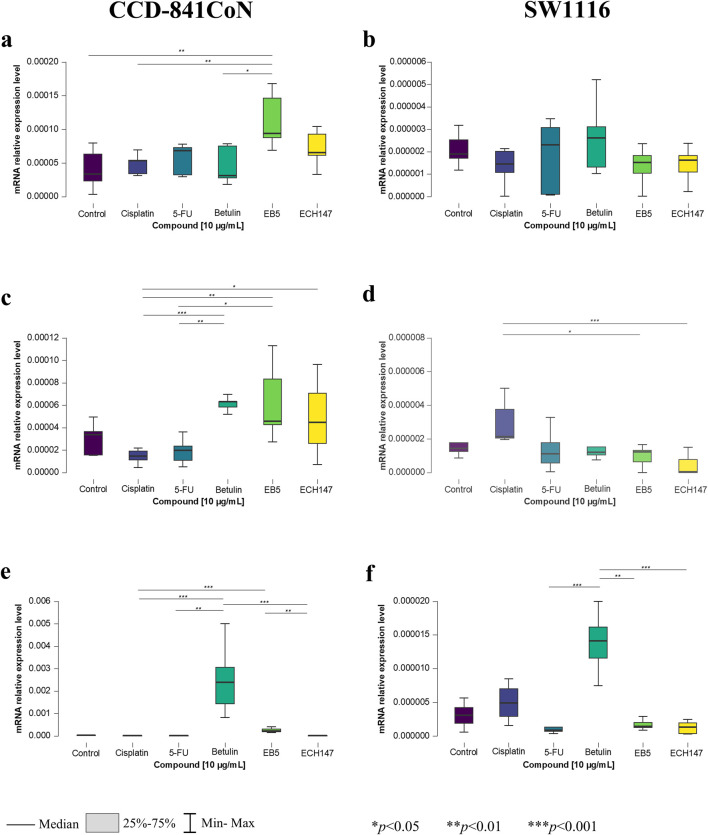
Changes in gene expression of *IL-6* in CCD-841CoN and SW1116 cell lines after 2 h **(a,b)**, 8 h **(c,d)** and 24 h **(e,f)** of exposure to the tested compounds. 5-FU—5-fluorouracil; EB5—28-propynoylbetulin; ECH147—29-diethoxyphosphoryl-28-propynoylbetulin; *—*p* < 0.05; **—*p* < 0.01; ***—*p* < 0.001.

To evaluate the temporal dynamics of *IL-6* gene expression under the influence of the tested compounds, real-time RT-qPCR analysis was performed on HT-29, RKO, SW1116, and CCD-841CoN cell lines. No *IL-6* gene expression was detected in HT-29 and RKO cells, either untreated or treated with the tested compounds, regardless of exposure time. In contrast, *IL-6* mRNA expression was observed in the SW1116 cell line—characterized by low-grade malignancy—as well as in the non-cancerous control line CCD-841CoN (Figure 1a).

After 2 h of incubation with the compounds, a statistically significant increase in *IL-6* expression was observed exclusively in CCD-841CoN cells (Figure 1a). Specifically, treatment with the EB5 derivative and betulin resulted in significantly higher expression compared to untreated controls (*p* < 0.05), and compared to cisplatin-treated cells (*p* < 0.01). In the SW1116 line, although a slight increase in *IL-6* expression after betulin treatment was observed after 2 h, the changes were not statistically significant (Figure 1b).

In the SW1116 line, *IL-6* expression increased significantly 8 h post-treatment with cisplatin compared to treatment with EB5 and ECH147 derivatives (*p* < 0.05 and *p* < 0.001, respectively) (Figure 1d). In CCD-841CoN cells, statistically significant differences were observed following treatment with betulin and its derivatives compared to reference compounds (Figure 1c). Treatment with betulin led to a notable increase in *IL-6* expression compared to cisplatin and 5-FU (*p* < 0.001 and *p* < 0.01, respectively). Furthermore, the EB5 derivative continued to induce *IL-6* expression after 8 h, with statistically significant differences observed in comparison to 5-FU (*p* < 0.05) and cisplatin (*p* < 0.01). Among the tested compounds, the smallest increase in *IL-6* expression was noted following treatment with the ECH147 derivative; nonetheless, a significant difference was found between ECH147 and cisplatin treatments (*p* < 0.05).

After 24 h of treatment, a shift in *IL-6* expression patterns in SW1116 cell line was observed (Figure 1f). A substantial increase in *IL-6* mRNA levels was detected following betulin treatment compared to EB5 (*p* < 0.01), ECH147 (*p* < 0.001), and 5-FU (*p* < 0.001). Notably, a marked decrease in *IL-6* expression was observed after 24 h of exposure to the derivatives, comparable to levels seen after 5-FU treatment at the same time point. A similar trend was also observed in CCD-841CoN cells (Figure 1e). Statistically significant increases in expression were observed following betulin treatment compared to 5-FU (*p* < 0.01), cisplatin, and ECH147 (*p* < 0.001). Additionally, a significant decrease in *IL-6* expression was noted after ECH147 treatment compared to EB5 (*p* < 0.001).

### 3.2 Effect of varying exposure times to investigated compounds on *IL-6* transcription

**FIGURE 2:**
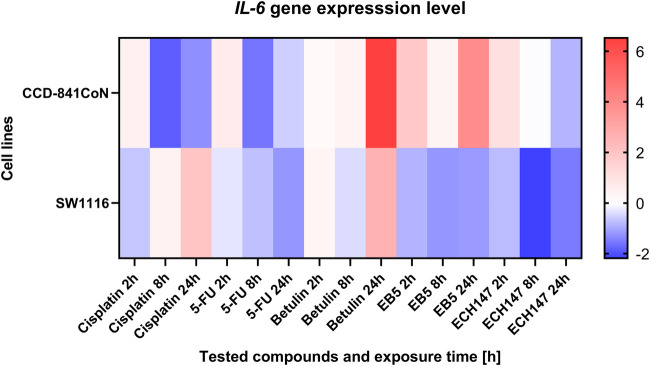
Heatmap illustrating the profile of *IL-6* gene expression in CCD-841CoN and SW1116 cell lines at different exposure times to the compounds. The scale represents log2FC. 5-FU—5-fluorouracil; EB5—28-propynoylbetulin; ECH147—29-diethoxyphosphoryl-28-propynoylbetulin; Red—indicates an increase in expression level compared to the control; Blue—indicates a decrease in expression level compared to the control.

To more effectively illustrate the variability in *IL-6* expression in the SW1116 and CCD-841CoN cell lines, a heatmap was generated depicting the cellular responses to the tested compounds, alongside their respective exposure durations. The expression results are shown as log2 fold changes (log2FC) compared to untreated control samples. To highlight differences in *IL-6* expression among the cell lines over various time points and treatments, a heatmap was created to illustrate the relative expression patterns (Figure 2).

Among the reference compounds, cisplatin and 5-FU exhibited similar patterns of *IL-6* expression depending on the duration of exposure. In all cases, the control cell line demonstrated a gradual decrease in *IL-6* expression over the course of treatment. Notably, the SW1116 cancer cell line showed the most pronounced response to 5-FU, with a continuous decline in *IL-6* levels throughout the exposure period.

In contrast, treatment with betulin led to an increase in *IL-6* expression across all tested cell lines, regardless of their origin, and this upregulation intensified with longer exposure times. A significant upregulation in *IL-6* mRNA expression was particularly evident 24 h post-treatment with betulin in both the SW1116 cancer cells and the normal CCD-841 cell line.

Regarding the betulin derivatives EB5 and ECH147, the SW1116 cancer cells consistently exhibited a reduction in *IL-6* gene expression at all examined time points. The most substantial decrease was observed 8 h after treatment with these derivatives. In the normal CCD-841 cell line, the downregulation of the *IL-6* expression was transient following exposure to EB5. However, treatment with ECH147 resulted in a sustained decrease of *IL-6* levels in both normal and cancerous cells, with a notably stronger reduction observed in the SW1116 cancer cell line.

These findings suggest that the betulin derivative ECH147 may possess a more favorable immunomodulatory profile compared to betulin itself, highlighting its potential as a selective agent for targeting IL-6-mediated inflammatory pathways in CRC.

### 3.3 Temporal changes in IL-6 protein levels in culture supernatants of tested cell lines

**FIGURE 3:**
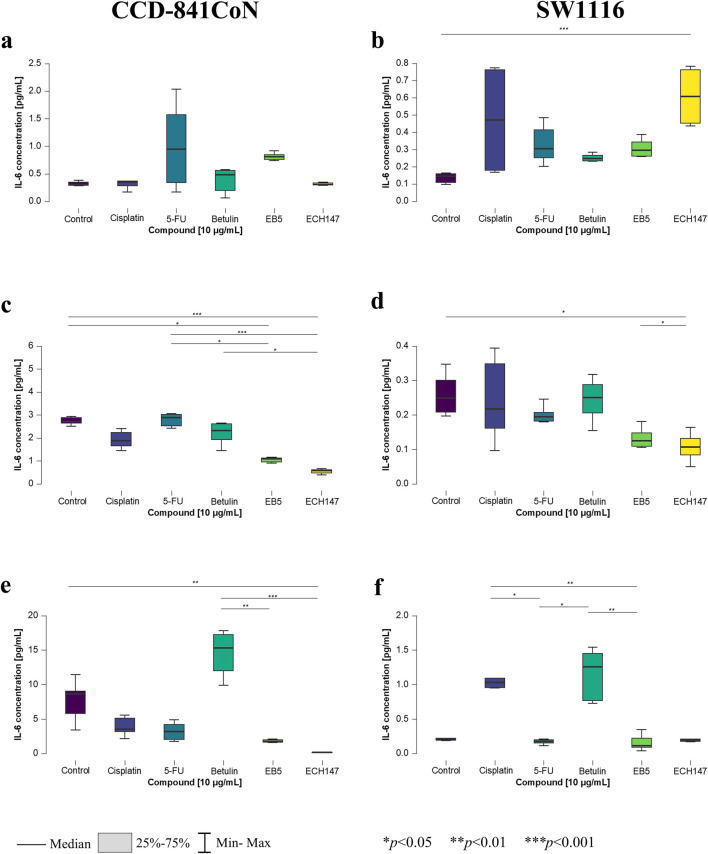
Changes in IL-6 protein concentration in cell culture medium in CCD-841CoN and SW1116 cell lines after 2 h **(a,b)**; 8 h **(c,d)** and 24 h **(e,f)** of exposure to the tested compounds. 5-FU—5-fluorouracil; EB5—28-propynoylbetulin; ECH147—29-diethoxyphosphoryl-28-propynoylbetulin; *—*p* < 0.05; **—*p* < 0.01; ***—*p* < 0.001.

To confirm the previously observed gene expression changes, the concentration of IL-6 protein secreted into the culture medium by the treated cells was also measured. For this purpose, a highly sensitive immunoassay was employed to detect subtle alterations in protein levels. Consistent with the gene expression data, no detectable IL-6 protein was observed in the culture medium of HT-29 and RKO cell lines. Interestingly, in the SW1116 and CCD-841CoN cell lines—where significant changes in *IL-6* mRNA expression had been reported—statistically significant alterations in IL-6 protein levels were also detected (Figure 3).

After 2 h of treatment, no statistically significant changes were observed in the concentration of IL-6 protein in the culture medium of the CCD-841CoN cell line (Figure 3a). In contrast, the SW1116 colorectal cancer cells exhibited a significant increase in IL-6 secretion upon treatment with the ECH147 derivative compared to untreated cells (*p* < 0.001; Figure 3b).

Significant alterations in IL-6 levels became apparent after prolonged compound exposure. After 8 h, untreated CCD-841CoN cells displayed a marked increase in IL-6 levels compared to cells treated with EB5 (*p* < 0.05) and ECH147 (*p* < 0.001; Figure 3c). Notably, IL-6 levels in cells treated with these derivatives remained stable over time. Moreover, the response of normal colon epithelial cells was consistent, showing elevated IL-6 secretion following 5-FU treatment in comparison to betulin derivatives, with changes comparable to the control group. Specifically, the increase in IL-6 levels relative to EB5 was statistically significant (*p* < 0.05), and even more pronounced when compared to ECH147 (*p* < 0.001). Additionally, betulin significantly stimulated IL-6 secretion compared to ECH147 (*p* < 0.05). In the SW1116 cancer cell line, a statistically significant decrease in IL-6 levels was observed in the culture medium following stimulation with ECH147 compared to both untreated cells and EB5-treated cells (*p* < 0.05; Figure 3d).

After 24 h of compound exposure, the observed changes in protein levels mirrored the trends previously reported at the mRNA level. A statistically significant reduction in IL-6 secretion was observed in CCD-841CoN cells treated with ECH147 compared to control (*p* < 0.01; Figure 3e). Moreover, betulin treatment led to an increased IL-6 secretion compared to both EB5 and ECH147 (*p* < 0.01 and *p* < 0.001, respectively). Interestingly, SW1116 cancer cells also showed increased IL-6 secretion following treatment with betulin relative to EB5 and 5-FU (*p* < 0.01 and *p* < 0.05, respectively). Furthermore, cisplatin treatment led to a significant increase in IL-6 protein levels compared to 5-FU (*p* < 0.05) and EB5 (*p* < 0.01) (Figure 3f).

### 3.4 Temporal regulation of IL-6 protein profile by tested compounds

**FIGURE 4:**
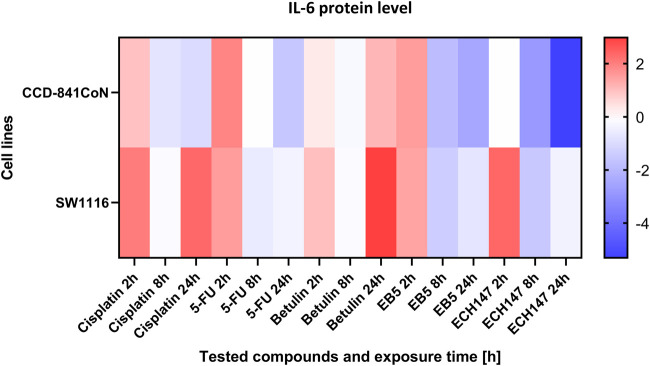
Heatmap illustrating the profile of IL-6 protein concentration changes in the CCD-841CoN and SW1116 cell lines at different exposure times to the compounds. The scale represents log2FC. 5-FU—5-fluorouracil; EB5—28-propynoylbetulin; ECH147—29-diethoxyphosphoryl-28-propynoylbetulin; Red—indicates an increase in expression level compared to the control; Blue—indicates a decrease in expression level compared to the control.

To visualize the dynamics of IL-6 secretion over time in response to compound treatment, a heatmap was generated based on the protein concentration data collected at multiple time points (Figure 4). This approach enabled a comparative assessment of the secretion profiles across different cell lines and treatment conditions. The heatmap clearly illustrates temporal trends in IL-6 release, highlighting both increases and decreases in cytokine levels depending on the duration of exposure and the specific compound applied. In the CCD-841CoN cell line, the highest levels of IL-6 protein secretion were observed 2 h after treatment with 5-FU and the EB5 derivative, as well as 24 h following treatment with betulin. Interestingly, the ECH147 derivative demonstrated a time-dependent reduction in IL-6 secretion, with the amount of protein released into the culture medium decreasing progressively with longer exposure. This downregulation was also observed in the SW1116 colorectal cancer cell line.

In SW1116 cells, a pronounced increase in IL-6 secretion was detected 24 h after treatment with both betulin and cisplatin. In contrast, treatment with 5-FU resulted in a decrease in IL-6 secretion after 8 h, with levels remaining relatively stable and low up to 24 h post-treatment. These findings indicate differential temporal regulation of IL-6 secretion depending on the compound and cell type, with betulin and cisplatin acting as potent inducers, while ECH147 and 5-FU appear to decrease IL-6 release over time.

### 3.5 Structural analysis of IL-6–ligand interactions with tested compounds

**FIGURE 5:**
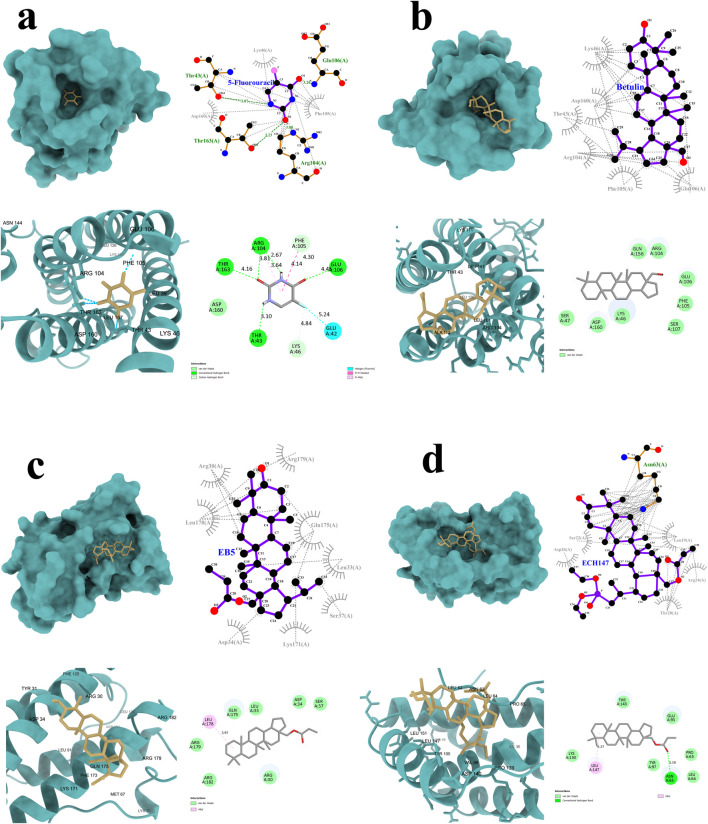
Graphical representation of the interactions between IL-6 and the tested compounds: 5-fluorouracil **(a)**, betulin **(b)**, EB5 derivative **(c)**, and ECH147 derivative **(d)**. Hydrogen bonds are highlighted in green.

To gain insight into the potential mechanisms of action of 5-FU (Figure 5a), betulin (Figure 5b), and its derivatives EB5 (Figure 5c) and ECH147 (Figure 5d), molecular docking studies were carried out. This *in silico* approach enables the prediction of molecular interactions between small molecules and target proteins, thereby supporting the identification of possible biological targets and guiding future drug design. The three-dimensional structure of the IL-6 protein was retrieved from the Protein Data Bank (PDB ID: 1ALU; https://www.rcsb.org/, accessed on 28 April 2025). Each compound was docked to the IL-6 structure, and the binding affinity was evaluated based on the calculated free binding energy (ΔG). A more negative ΔG value indicates a stronger and potentially more stable interaction between the ligand and the protein.

The results of the molecular docking simulations revealed that the strongest predicted interaction with IL-6 was observed for betulin, with a binding energy of −7.2 kcal/mol, while the weakest interaction was noted for 5-FU (−4.8 kcal/mol). Despite its high binding affinity, betulin did not form hydrogen bonds with the IL-6 protein. Instead, its interaction profile was dominated by numerous hydrophobic contacts and Van der Waals forces. A similar interaction pattern was observed for the EB5 derivative, which also did not form hydrogen bonds with the target protein.

In contrast, the ECH147 derivative formed one strong hydrogen bond with the Asn63 residue of IL-6. In addition, ECH147 exhibited hydrophobic interactions, Van der Waals interactions, and alkyl interactions with the protein, suggesting a more diverse binding mode. Although 5-FU showed the weakest overall binding affinity, it formed a network of four hydrogen bonds with the Thr43, Arg104, Glu106, and Thr163 residues of IL-6. Moreover, two additional C–H bonds may contribute to the stability of the complex. A complete summary of the molecular docking parameters and interaction types for all tested compounds is presented in Table 2.

**TABLE 2:** Comparative analysis of docking parameters for betulin, EB5, ECH147, and 5-FU with IL-6 protein.

Parameter	Betulin	EB5	ECH147	5-FU
Binding Energy [kcal/mol]	−7.2	−6.4	−5.6	−4.8
Hydrogen Bonds	0	0	1	4
Residues H-bonded	n/d	n/d	Asn63	Thr43; Arg104; Glu106; Thr163
Hydrophobic Interactions	6	8	5	3
Carbon–Hydrogen Bonds	0	0	0	2
Van der Waals Interactions	8	7	6	1
Alkyl Interactions	0	1	1	1

EB5—28-propynoylbetulin; ECH147—29-diethoxyphosphoryl-28-propynoylbetulin; 5-FU—5-fluorouracil.

## 4 Discussion

Chronic inflammation is a hallmark of CRC and plays a central role in its development and progression. As one of the most common malignancies of the gastrointestinal tract, CRC remains a major focus of research aimed at identifying alternative therapeutic strategies that could reduce reliance on conventional chemotherapy, which is often associated with significant side effects and patient burden. In this context, increasing attention has been directed toward targeting inflammatory mediators, with IL-6 emerging as a particularly promising candidate.

Elevated IL-6 expression has been linked to poor prognosis, therapy resistance, and advanced clinical stage in CRC patients (Zhou et al., 2025). These findings underscore the therapeutic potential of modulating IL-6-related pathways as part of a more targeted treatment approach.

Among the compounds investigated for their anti-inflammatory and anticancer potential, pentacyclic triterpenes—such as betulin—have shown encouraging results. However, the clinical application of betulin is limited due to its poor solubility and low bioavailability. To overcome these challenges, synthetic derivatives such as EB5 and ECH147 have been developed to enhance its pharmacokinetic and pharmacodynamic properties, potentially making them more effective therapeutic agents.

The study aimed to evaluate the ability of betulin and its derivatives (EB5 and ECH147) to modulate IL-6 expression in CRC. To this end, we investigated their effects at both the mRNA and protein levels in three CRC cell lines—SW1116, HT-29, and RKO—which differ in clinical aggressiveness from least to most aggressive (Ahmed et al., 2013). Normal colon epithelial cells (CCD-841CoN) were also included to assess selectivity. Furthermore, the time dependency of the response was analyzed by measuring IL-6 at mRNA and protein levels after 2, 8, and 24 h of treatment with each compound at a concentration of 10 μg/mL.

To evaluate changes in *IL-6* gene expression induced by the tested compounds, real-time RT-qPCR analysis was performed. Notably, *IL-6* expression was absent in two CRC cell lines harboring the *BRAF* ^*V600E*^ mutation, HT-29 and RKO, whereas it was detectable in the normal colon epithelial cell line CCD-841CoN and in the CRC cell line SW1116, characterized by low malignancy and lacking BRAF mutations (Ahmed et al., 2013). The most pronounced reduction in *IL-6* mRNA levels was observed 8 h post-treatment with the ECH147 derivative in the SW1116 cell line. Similarly, the CCD-841CoN line also demonstrated a progressive decrease in *IL-6* expression over time relative to untreated controls. Treatment with the EB5 derivative elicited comparable, though less marked, downregulation of *IL-6*, predominantly in cancerous cells, with minimal effect on the normal cell line. Interestingly, both normal and cancerous cells exhibited a significant upregulation of *IL-6* expression 24 h after betulin treatment. The absence of *IL-6* expression in HT-29 and RKO tumor cells, in contrast to normal colonocytes, may reflect the fact that, in advanced stages of tumor development, IL-6 is primarily produced by cells within the tumor microenvironment and immune cell populations, rather than by the tumor cells themselves (Tong et al., 2022; Wang et al., 2024).

These findings are consistent with those reported by Janus et al. (Janus et al., 2022), who investigated the role of heat shock transcription factor 1 in suppressing overactivation of stress response genes (*ATF3*, *JUN*, *FOS*) and pro-inflammatory cytokines (*TNFα*, *IL-6*) in non-cancerous and RKO CRC cells. They did not observe IL-6 expression in RKO cells, but additionally, using CRISPR/Cas9-mediated *HSF1* knockout, they confirmed the independent role of this factor in *IL-6* transcription in these cells, which is consistent with our finding.

Team by Holmer et al. (2015) similarly reported absent endogenous *IL-6* expression in HT-29 cells. Their study examined the effects of classical and trans-signaling IL-6 pathways on CEACAM5 and CEACAM6 gene expression by RT-qPCR, revealing that despite the lack of IL-6, HT-29 cells exhibit high basal levels of *CEACAM5* and *CEACAM6*, which were further enhanced by IL-6 trans-signaling. The authors proposed that *CEACAM5* and *CEACAM6* expression may be regulated independently of classical IL-6 signaling, potentially through alternative mechanisms such as HIF-1α stabilization. Our data corroborate these findings, showing persistent absence of *IL-6* expression in HT-29 and RKO cells even after treatment, implying that the tested compounds act through IL-6/sIL-6R-independent pathways in these lines.

In a clinical context, Malicki et al. (2009) reported elevated IL-6 and IL-8 levels in blood and tissues of CRC patients at both mRNA and protein levels, which decreased after tumor resection. Similarly, our study showed IL-6 expression in normal CCD-841CoN and SW1116 CRC cells. Treatment with ECH147 downregulated IL-6, suggesting a potential anti-tumor effect by decreasing of IL-6-related pathways involved in CRC metastasis.

The effects of betulin and its alkynyl derivatives on inflammation-related gene expression were also explored by Lubczyńska et al. (Lubczyńska et al., 2023). Their study employed the HT-29 cell line utilizing HG-U133A oligonucleotide microarrays to profile changes in genes associated with cytokine-mediated signaling pathways. We observed that the influence of betulin on *IL-6* mRNA levels varied depending on the specific chemical modifications of the derivatives as well as the cellular context. Although betulin induced significant alterations in *IL-6* expression across multiple time points, the response differed between cell lines, indicating that structural changes to the betulin molecule may distinctly affect IL-6 signaling pathways.

To confirm the observed mRNA-level changes, protein expression in the cell culture medium was analyzed using the PLA. Consistent with the transcript-level data, no IL-6 protein expression was detected in the RKO and HT-29 cell lines. In contrast, IL-6 was present in both the SW1116 colorectal cancer cells and the non-cancerous CCD-841CoN colon epithelial cells. Among the tested compounds, the ECH147 derivative led to the most pronounced reduction in IL-6 protein levels after 24 h of treatment in both healthy and cancerous cells. A similar, though less significant, effect was observed following treatment with the EB5 derivative.

In our previous study (Madej et al., 2025), where we reported that ECH147 most effectively downregulated IL-8 expression at both mRNA and protein levels in CRC cell lines we observed a similar effect. The study was concluded that the expression profile of IL-8 may vary depending on the malignancy of the cell line and its genetic background (e.g., *BRAF* mutation), which in turn influences compound efficacy. Nevertheless, ECH147 consistently reduced IL-8 expression across all tested cell lines. This suggests that ECH147 may simultaneously downregulate both IL-6 and IL-8 expression, regardless of genetic status. Importantly, the dual downregulation of IL-6 and IL-8 by ECH147 suggests that this compound may exert broader anti-inflammatory and anti-tumour effects by modulating key pro-inflammatory cytokines and their associated signaling pathways or via long non-coding RNA (lncRNA)– microRNA (miRNA) networks that coordinate cytokine expression, as described by Xiao et al. (Xiao et al., 2020).

These results are in line with those of Wang et al. (2019), who used Western blot and ELISA to measure IL-6 protein levels in CRC cell lines DLD1 and HT-29. They also reported a lack of IL-6 secretion by these cells, consistent with our findings. Furthermore, they demonstrated that increased *FRA1* expression correlated with IL-6 secretion and poorer patient prognosis. This supports the hypothesis that lower IL-6 expression may be associated with improved clinical outcomes. The differential IL-6 expression observed across CRC cell lines with distinct genetic backgrounds underscores the possible influence of tumor mutational landscape on cytokine-mediated inflammatory responses, consistent with evidence implicating tumor suppressor gene mutation burden in cancer susceptibility and progression (Ning et al., 2023).

Similar conclusions were drawn by Zeng et al. (2017), who used immunohistochemistry to assess IL-6 expression levels in colorectal tumor tissues compared to adjacent healthy tissue. Their study showed that elevated IL-6 levels correlated with advanced CRC clinical stage and suggested IL-6 as a potential biomarker. In our study, we also observed that SW1116 colorectal cancer cells exhibited higher IL-6 protein levels than non-cancerous cells, and that treatment with ECH147 significantly reduced this expression. This finding underscores the potential of ECH147 as a candidate for therapeutic development targeting IL-6.

To further investigate the mechanism of action, molecular docking analyses were performed. Among all tested compounds, only ECH147 was found to form stable hydrogen bonds with IL-6. Taken together, these results suggest that ECH147 exhibits the strongest anti-inflammatory activity among the compounds tested, possibly through direct interaction with the IL-6 protein in SW1116 and CCD-841CoN cells. Nevertheless, additional experimental approaches and validation are required to confirm this mechanism of action at the molecular level (Zhang et al., 2022).

In a study conducted by Peng et al., the authors investigated the role of proteasome 26S subunit non-ATPase 12 (PSMD12) in hepatocellular carcinoma (HCC), focusing on its ability to stabilize cyclin-dependent kinase 1 (CDK1). Using a combination of bioinformatic analyses and experimental approaches based on cell culture and xenograft models, they demonstrated that PSMD12 promotes HCC progression by influencing the G2/M phase of the cell cycle and modulating the PI3K/AKT/mTOR signaling pathway. The authors concluded that PSMD12 plays a crucial role in regulating CDK1 activity in HCC cells (Peng et al., 2025). Moreover, the observed high expression levels of both genes and their positive correlation suggest a functional association. In addition, the identification of the CDK1/PLK1/AKT signaling axis, regulated by PSMD12, indicates that this protein may participate in the control of mitotic progression and the activation of oncogenic signaling mechanisms (Peng et al., 2025). Our study was also based on a similar integrative approach combining bioinformatic and experimental methods. The compound ECH147, which most strongly inhibited IL-6 expression at both the mRNA and protein levels, also exhibited high stability when forming a complex with IL-6. This finding suggests that the ECH147 derivative may influence one or more stages of the IL-6/JAK/STAT3 signaling pathway; however, further studies are required to elucidate its precise role in downstream regulatory processes.

Currently, several inhibitors available on the market act either by blocking the binding site of IL-6 to its receptor IL-6R together with the gp130 subunit, or by forming stable complexes directly with the IL-6 protein itself. The study by Parisi et al. presented the latest findings concerning Tocilizumab (TCZ). TCZ is a monoclonal antibody specifically designed to block the IL-6 binding site on the IL-6 receptor (IL-6R). Its mechanism of action involves inhibition of both the classical and trans-signaling pathways of IL-6, and it is mainly used in the treatment of rheumatoid arthritis (Parisi et al., 2025). Another example is sarilumab, which acts in a similar manner to TCZ by binding to the IL-6R receptor and preventing IL-6 from attaching to it (Wang et al., 2025). A third example is siltuximab, which is indicated for the treatment of multicentric Castleman’s disease. Siltuximab is a chimeric monoclonal antibody with high affinity and strong binding to the IL-6 protein, resulting in a reduction of its bioactivity (Chen and Chen, 2015).

In our studies, we observed a potential similar mechanism of action, particularly for the phosphonate derivative of betulin, designated as ECH147. Incorporation of this substituent into the betulin structure enhanced the strength and stability of IL-6 binding, which may have led to decreased IL-6 activity and a reduction in IL-6 levels during the exposure of CRC cells to the tested compound. Additionally, the observed decrease in *IL-6* mRNA expression may indicate a potential mechanism by which ECH147 interacts with the IL-6 receptor or its subunit, ultimately leading to the suppression of IL-6 expression through inhibition of the IL-6/JAK/STAT3 signaling pathway. Therefore, we suggest that future research should focus on the effects of the tested compounds on the components of this pathway, as well as on the IL-6 receptor itself, as a potential molecular target of the ECH147 derivative in CRC.

In conclusion, our data indicate that phosphonate derivative ECH147 is the most effective of the tested compounds in downregulating IL-6 expression in CRC cells. Its effect on IL-6 expression, suggests promising potential as a targeted anti-inflammatory agent in CRC. Future studies should focus on validating these effects in advanced *in vitro* models, such as 3D cell cultures that better recapitulate the tumor microenvironment, and on exploring the integration of these compounds into advanced drug delivery platforms, such as polymer-based nanoparticles, liposomes, or other nanomaterials, as well as stimuli-responsive carriers, to enhance their selectivity, bioavailability, and clinical applicability Additionally, he chemistry-focused perspective on phosphorylation and alkynyl modifications, which enhance the activity of betulin derivatives, may also prove relevant for other chemical compounds undergoing similar modifications, aligning well with recent advances in synthetic chemistry (Hong et al., 2023; Olakowska et al., 2022; Qin et al., 2024).

### 4.1 Limitations and future directions

CRC therapy still relies primarily on surgical intervention, often combined with chemotherapy, which is associated with high systemic toxicity. Immunotherapy remains applicable only in selected cases. Due to the limited efficacy of current treatments, increasing attention is directed toward identifying novel bioactive compounds or applying drug repurposing strategies, which enable the use of known molecules in new therapeutic contexts, reducing both development time and cost (Gila et al., 2025).

Betulin, a naturally occurring compound with well-documented antibacterial, antiviral, and anti-inflammatory properties, represents one such promising molecule (Serrafi and Wasilewski, 2025). However, its poor bioavailability limits its direct therapeutic use. Consequently, various chemical modifications have been undertaken to obtain derivatives with improved pharmacological and pharmacokinetic properties. In the present study, two such derivatives—EB5 and ECH147 — were investigated. Although scientific data on these compounds remain scarce, previous reports and current findings indicate their potential to modulate interleukin-related signaling pathways (IL-6 and IL-8), which play key roles in inflammation and colorectal carcinogenesis (Madej et al., 2025).

One limitation of this study is the exclusive use of two-dimensional (2D) cell culture models, which, despite enabling analysis at the single-cell level, fail to reproduce the complex tumor microenvironment. Therefore, future research should employ three-dimensional (3D) models such as spheroids and patient-derived organoids, as well as a broader range of cell lines, to better mimic physiological tumor conditions (Hanitrarimalala et al., 2025; D'Angelo et al., 2026). The study also provides insights into the effects of EB5 and ECH147 on IL-6 expression, suggesting possible modulation of signaling pathways such as JAK/STAT3, MAPK/ERK, and PI3K/AKT (Tong et al., 2022). Further analyses are necessary to confirm these effects and clarify molecular mechanisms. *In vivo* studies are also required to characterize pharmacokinetic and pharmacodynamic (PK/PD) profiles and to explore advanced formulations, including nanoparticle conjugates, which could enhance delivery and bioavailability. Preliminary molecular docking analyses revealed favorable binding affinities of both ligands to selected protein targets, forming a foundation for further *in silico* investigations (Xie et al., 2025; Zhang et al., 2022).

**FIGURE 6:**
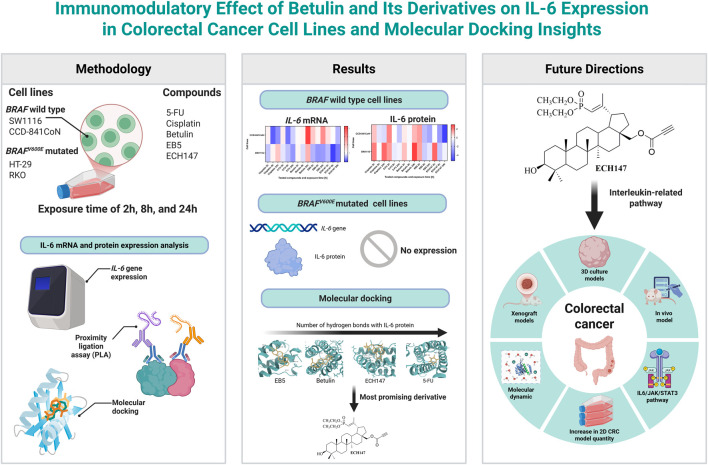
Integrative summary of current findings and future research directions. Created in BioRender. Madej, M. (2025) https://BioRender.com/llz7yca.

In summary, EB5 and ECH147 demonstrate promising biological activity and represent valuable candidates for continued studies employing 3D culture systems, *in vivo* models, and mechanistic analyses, potentially contributing to the development of novel anti-inflammatory strategies in CRC therapy (Figure 6).

## 5 Conclusion

This study demonstrates that betulin and its synthetic derivatives, EB5 and ECH147, differentially modulate IL-6 expression in colorectal cancer (CRC) and normal colon epithelial cells. Among the tested compounds, ECH147 exhibited the most consistent and pronounced inhibitory effect on IL-6 expression at both the mRNA and protein levels, particularly in SW1116 cancer cells and CCD-841CoN normal cells. These effects were time-dependent and correlated with molecular docking results, which indicated stable interactions between ECH147 and the IL-6 protein via hydrogen bonding. Importantly, CRC cell lines harboring the *BRAF* ^*V600E*^ mutation (HT-29 and RKO) did not exhibit IL-6 expression at either the mRNA or protein level, regardless of compound treatment or exposure time. This suggests that IL-6 expression in CRC may be strongly influenced by the underlying genetic background, and that *BRAF*-mutated tumors may be less responsive to IL-6-targeted interventions. Therefore, this factor should be considered in the design of new therapeutic strategies for CRC, which ought to be tailored to individual patients given the heterogeneous nature of this disease. Overall, our findings support the therapeutic relevance of targeting IL-6 signaling pathways using chemically modified triterpenes and suggest that ECH147 may serve as a promising lead compound for further preclinical investigation.

## Data Availability

The original contributions presented in the study are included in the article/supplementary material, further inquiries can be directed to the corresponding author.
